# The use of positron emission tomography/computed tomography imaging in radiation therapy: a phantom study for setting internal target volume of biological target volume

**DOI:** 10.1186/s13014-014-0315-2

**Published:** 2015-01-08

**Authors:** Wataru Kawakami, Akihiro Takemura, Kunihiko Yokoyama, Kenichi Nakajima, Syoichi Yokoyama, Kichiro Koshida

**Affiliations:** Department of Radiological Technology, Public Central Hospital of Matto Ishikawa, 3-8, Kuramitsu, Hakusan City, Ishikawa Pref 924-8588 Japan; Department of Quantum Medical Technology, Division of Health Sciences, Graduate School of Medical Science, Kanazawa University, Ishikawa, Japan; PET Imaging Center, Public Central Hospital of Matto Ishikawa, Ishikawa, Japan; Department of Nuclear Medicine, Kanazawa University Hospital, Ishikawa, Japan

**Keywords:** PET/CT, BTV, Respiratory motion, ITV, Attenuation correction

## Abstract

**Background:**

Fluorodeoxyglucose (^18^F-FDG) positron emission tomography/computed tomography (PET/CT) is an important method for detecting tumours, planning radiotherapy treatment, and evaluating treatment responses. However, using the standardized uptake value (SUV) threshold with PET imaging may be suitable not to determine gross tumour volume but to determine biological target volume (BTV). The aim of this study was to extract internal target volume of BTV from PET images.

**Methods:**

Three spherical densities of ^18^F-FDG were employed in a phantom with an air or water background with repetitive motion amplitudes of 0–30 mm. The PET data were reconstructed with attenuation correction (AC) based on CT images obtained by slow CT scanning (SCS) or helical CT scanning (HCS). The errors in measured SUV_max_ and volumes calculated using SUV threshold values based on SUV_max_ (TH_max_) in experiments performed with varying extents of respiratory motion and AC were analysed.

**Results:**

A partial volume effect (PVE) was not observed in spheres with diameters of ≥ 28 mm. When calculating SUV_max_ and TH_max_, using SCS for AC yielded smaller variance than using HCS (*p* < 0.05). For spheres of 37- and 28-mm diameters in the phantom with either an air or water background, significant differences were observed when mean TH_max_ of 30-, 20-, or 10-mm amplitude were compared with the stationary conditions (*p* < 0.05). The average TH_max_ values for 37-mm and 28-mm spheres with an air background were 0.362 and 0.352 in non-motion, respectively, and the mean TH_max_ values for 37-mm and 28-mm spheres with a water background were 0.404 and 0.387 in non-motion and 0.244 and 0.263 in motion, respectively. When the phantom background was air, regardless of sphere concentration or size, TH_max_ was dependent only on motion amplitude.

**Conclusions:**

We found that there was no PVE for spheres with ≥ 28-mm diameters, and differences between SUV_max_ and TH_max_ were reduced by using SCS for AC. In the head-and-neck and the abdomen, the standard values of TH_max_ were 0.25 and 0.40 with and without respiratory movement, respectively. In the lungs, the value of TH_max_ became the approximate expression depending on motion amplitude.

## Background

Positron emission tomography/computed tomography (PET/CT) has become an important and commonly used imaging method to detect tumours, plan radiotherapy treatment, and evaluate responses to therapy, as it allows for simultaneous functional and anatomical imaging. During treatment planning for radiation therapy, gross tumour volume (GTV) is calculated based on CT images. Moreover, it has been reported that when planning treatment for tumours of the head-and-neck, inter- and intra-observer differences in GTV may be reduced by using PET images [1].

There are many issues that arise when determining GTV with only PET imaging, such as accounting for respiratory movement and distinguishing inflammation. PET imaging also involves limited spatial resolution and the partial volume effect (PVE). Moreover, it has been reported that extraction volume differs according to the standardized uptake values (SUV) of normal tissue and tumour [2]. Thus, it is not suitable to automatically extract GTV using the SUV from PET images. Realistically, it is preferable that the radiotherapist uses the PET images as a reference [3].

Given that the PET image was a function image, the extracted volume is thought to be biological target volume (BTV). BTV often indicates the part with especially high tumour revitalization in GTV [4]. It became possible to irradiate GTV and BTV by a separate dose by the development of the radiation therapy machine and the plan device. Therefore, BTV is being used for radiation therapy [5].

Several research reports suggest extracting BTV from PET images [6-12]. However, a concrete extraction condition that tempers with various situations has not been established to date. The purpose of this study was to obtain conditions to automatically extract internal target volume of BTV (ITV-b) from phantom experiments using SUV threshold values based on SUV_max_ (TH_max_). The items that should be considered for extracting ITV-b using TH_max_ are as follows: (1) tumour size (PVE), (2) motion amplitude, (3) tumour site (lung or head-and-neck, abdomen), and (4) attenuation correction (AC). We examined tumour sizes for which a PVE was not observed [13,14] in addition to changes in SUV_max_ and TH_max_ according to the degree of motion amplitude [15-17], the position of the tumour [18], and the method of attenuation correction [19-21].

## Methods

### The National Electrical Manufacturers Association (NEMA) 2001 International Electrotechnical Commission (IEC) Phantom

In this study, we imaged a PET image quality phantom (NEMA/IEC phantom; Data Spectrum Corp.). This phantom contained six hollow spheres with internal diameters of 10, 13, 17, 22, 28, and 37 mm, inside a simulated body cavity that may be filled with water. The phantom background was filled with either water or air. The phantom background was filled with water to simulate a human head-and-neck, abdominal and pelvic tumour. In this series of experiments, all spheres were filled with 2-[fluorine-18] fluoro-2-deoxy-D-glucose (^18^F-FDG) solution that was measured in a dose calibrator and diluted with water to obtain a radioisotope concentration (RC) of 16–64 kBq/mL. The total weight of the filled phantom was 12.4 kg. The phantom background was filled with air to simulate a human lung tumour. In this series of experiments, all spheres were filled with ^18^F-FDG solution diluted with water to obtain an RC of 64–256 kBq/mL. The total weight of the phantom in this situation was 2.4 kg.

The phantom background was filled with water or air, yielding sphere-to-background (S/B) ratios of approximately 4, 8, and 16 to 1 or null. To achieve the same S/B ratio, each given dose was adjusted by the difference of the background. The RC values of the spheres and background and the S/B ratios were chosen to simulate the ranges obtained from clinical conditions based on the tumour concentration of ^18^F-FDG and the SUV. The SUV is commonly used as a relative measure of FDG uptake. The basic formula for SUV was as follows:

1 $$ \mathrm{S}\mathrm{U}\mathrm{V}=\frac{\mathrm{C}}{\left(\mathrm{A}/\mathrm{W}\right)}, $$

where C was the RC (kBq/mL) measured by the PET scanner within the region of interest, A was the amount of injected activity (kBq), and W was the weight of the patient (g).

A motion system (QRP-01; Qualita, Ageo City, Saitama Prefecture, Japan) was used in this study to simulate respiratory motion. The NEMA/IEC phantom was placed on a moving table. The moving table was oscillated with a displacement of 10, 20, and 30 mm in the cranio-caudal direction at a frequency of 15 cycles/min. These parameters were selected to simulate displacements and respiratory cycles typically observed in normal respiratory motion.

### Imaging

CT transmission (for AC) and PET scans were performed using a PET/CT scanner (Discovery PET/CT 600 Motion; General Electric Medical Systems [GEMS], Milwaukee, WI, USA) with a stationary or moving phantom. Scans of the moving phantom were acquired without gating. Slow CT scanning (SCS; 120 kV, 45 mA, and 4.0 s/rotation) or the helical CT scanning (HCS; 120 kV, 360 mA, 0.5 s/rotation, and pitch 0.938) imaging data, followed by PET emission data, were acquired for 300 s from one field of view. Both PET scan types (stationary and motion) were acquired in 3-dimensional (3D) mode. The 3D data were first Fourier rebinned, and then all emission scans were reconstructed into a 256 matrix using ordered-subset expectation maximization algorithms. The numbers of iterations and subsets were 2 and 32, respectively. The PET-reconstructed slice thickness was 3.75 mm.

### Phantom object

The NEMA/IEC phantom, which contained six spheres ranging from 10 to 37 mm in diameter, was filled with ^18^F-FDG. PET/CT data acquired with the stationary phantom was used as a standard (non-motion). Respiratory motion was simulated by a motor-driven plastic platform that moved the phantom with a displacement of 10, 20, and 30 mm in the cranio-caudal direction at a frequency of 15 cycles/min. A 4-s period of oscillation was selected based on the average respiratory period measured in 50 patients with lung tumours at our centre using a respiratory gating system (Real-time Positioning Management System; Varian, Palo Alto, CA, USA). With the phantom in motion, PET data were acquired in the static mode, wherein data were collected for a definite time period according to the width of the detector’s body axis instead of continuous movement like in CT. Acquisitions using three types of S/B ratios, four different motion amplitudes, two different background media, and two types of CT scans were repeated three times; for a total of 144 image acquisitions performed.

### Data analysis

**Figure 1:**
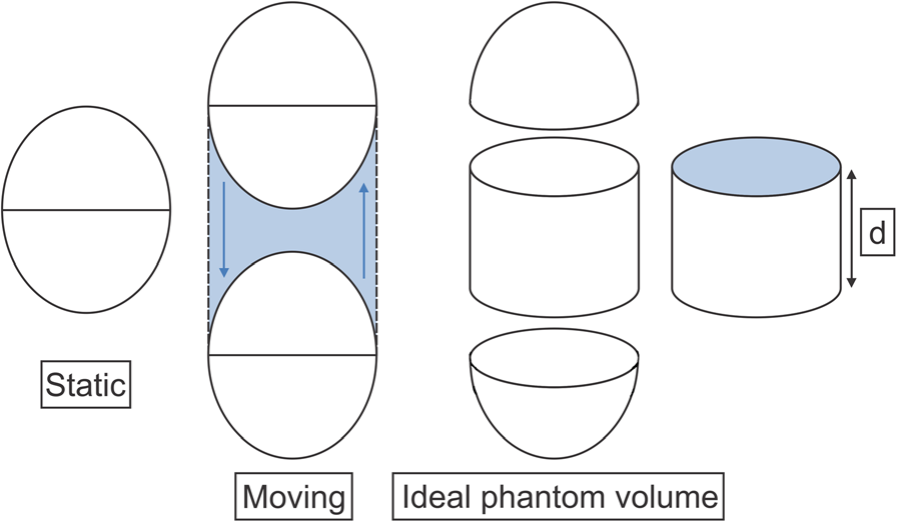
**Diagram depicting the volumes of stationary and moving spherical phantoms.** The ideal phantom volumes were divided into a sphere and a column, and each volume was calculated. d, motion amplitude.

Image analysis was performed using PET VCAR™ software and the Advantage Workstation (GE Healthcare, Milwaukee, WI, USA). This software is designed to measure SUV and extracted volume for any PET-defined region with detected activity. SUV_max_ values were measured in spheres with 144 image acquisitions. The SUV threshold values based on SUV_max_ (TH_max_) were calculated so that the volume extracted by the TH_max_ matched the ideal volume of the spherical tumours in the phantom, taking into consideration the motion amplitude. Thus, an ideal phantom volume becomes a total of the volume of spheres and the volume of the column corresponding to the amount of the amplitude (Figure 1).

### Evaluation of PVE

To correctly assess the influence of respiratory movement, it is necessary to use a volume that will not be affected by the PVE. Therefore, the PVE was evaluated using the data from scans with the stationary phantom, with the recovery coefficient (RCo) calculated as follows:

2 $$ \mathrm{R}\mathrm{C}\mathrm{o}=\mathrm{Ci}/\mathrm{C}\mathrm{r}, $$

where Ci was the maximum radioactivity of each sphere at each S/B ratio, and Cr was the maximum radioactivity of the 37-mm sphere at each S/B ratio in non-motion, both measured in scans with no phantom motion. Evaluation was performed using two types of background (air and water) and attenuation corrections (SCS and HCS). An analysis of variance (ANOVA) was used to check for significant differences in the sphere diameter. Next, multiple comparisons between six types of spheres of (10–37 mm) were performed using Dunnett’s method and the size without the influence of PVE was then examined.

### Analysis of SUV_max_ and TH_max_

Effects of sphere size, motion amplitude, background medium, and attenuation correction on measured SUV_max_ and TH_max_ were examined. The spheres with 37-mm and 28-mm diameters were used for this evaluation. The phantom, with backgrounds of both air and water, were scanned with the SCS and HCS, as described above. During scanning, the phantom was moved with amplitudes of 0, 10, 20, or 30 mm. To evaluate the influence of all combined S/B ratios on SUV_max_, the ratios of SUV_max_ (SUVr) were calculated using the average SUV_max_ for the three S/B ratios in scans performed with no phantom motion.

The SUVr was calculated as follows:

3 $$ \mathrm{SUVr}=\mathrm{C}\mathrm{j}/\mathrm{C}\mathrm{a}, $$

where Cj was the average of SUV_max_ values obtained for all S/B ratios with a particular sphere size, and Ca was the average of SUV_max_ values obtained for all S/B ratios with the same sphere size and no phantom motion. The variances in SUVr were compared between the attenuation corrections using the *F*-test. The differences between the mean SUVr obtained with different motion amplitudes were evaluated using ANOVA with Dunnett’s multiple comparisons.

The variances of TH_max_ were compared with the attenuation corrections using the *F*-test. The variations of the mean TH_max_ with different amplitudes were evaluated by ANOVA and Dunnett’s multiple comparisons.

## Results

### Partial volume effects

Table 1 shows the RCo values measured in our study. A significant difference was observed between RCo values measured in spheres of different sizes with phantoms using either air or water as background, scanned with SCS or HCS. When evaluating all RCo values measured using each of the CT methods and both of the background media used, no PVE influence on RCo was observed in spheres with diameters of 28 mm or larger. Conversely, we demonstrated that the spheres with diameters of 22 mm or less might be affected by PVE. Therefore, we only analysed 28- and 37-mm spheres in subsequent experiments.

**Table 1:** **Recovery coefficients by sphere size**

**Sphere size (mm)**		**Background**			
		**Air**		**Water**	
		**SCS**	**HCS**	**SCS**	**HCS**
		**Mean ± SD**	**Mean ± SD**	**Mean ± SD**	**Mean ± SD**
Rco	37	1.00 ± 0.01	1.00 ± 0.01	1.00 ± 0.03	1.00 ± 0.02
	28	0.99 ± 0.01	0.99 ± 0.01	1.00 ± 0.04	0.98 ± 0.02
	22	0.98 ± 0.01**	0.98 ± 0.01**	0.95 ± 0.03	0.93 ± 0.04**
	17	0.91 ± 0.01**	0.90 ± 0.01**	0.91 ± 0.06**	0.87 ± 0.06**
	13	0.80 ± 0.02**	0.79 ± 0.01**	0.74 ± 0.06**	0.76 ± 0.04**
	10	0.56 ± 0.02**	0.56 ± 0.02**	0.57 ± 0.04**	0.56 ± 0.05**

**indicates *p* < 0.05 compared with the 37-mm sphere using Dunnett’s multiple comparisons.

Rco, recovery coefficient; SCS, slow computed tomography scanning; HCS, helical computed tomography scanning; SD, standard deviation.

### SUV_max_

**Figure 2:**
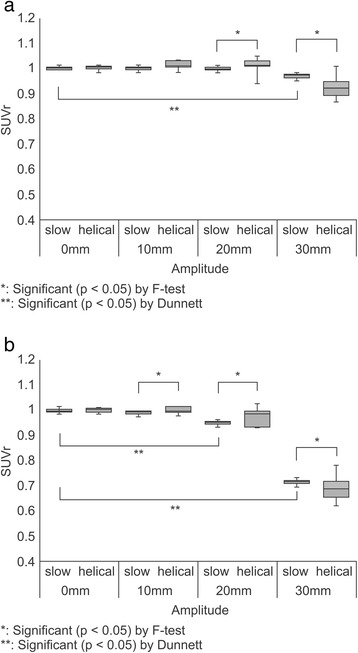
**Effect of motion amplitude on maximum standard uptake value ratio (SUVr), evaluated with air as the phantom background. (a)** The variance of SUVr with slow computed tomography (CT) scanning (SCS) was significantly smaller than that of SUVr with helical CT scanning (HCS) for a 37-mm sphere with 20- and 30-mm amplitude (*F*-test: *p* < 0.05). A significant difference was observed when comparing the mean SUVr between 30-mm amplitude and stationary conditions (Dunnett’s test: *p* < 0.05). **(b)** The variance of SUVr with SCS was significantly smaller than that of SUVr with HCS for a sphere of 28 mm with 10-, 20-, and 30-mm motion amplitudes (*F*-test: *p* < 0.05). Significant differences were observed when comparing the mean SUVr of 30- and 20-mm motion amplitudes with stationary conditions (Dunnett’s test: *p* < 0.05).

**Figure 3:**
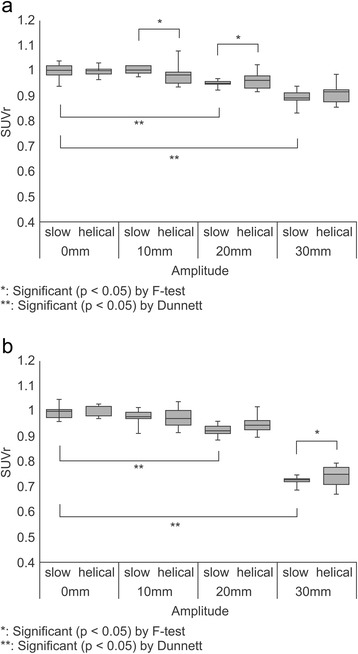
**Effect of motion amplitude on maximum standard uptake value ratio (SUVr), evaluated with water as the phantom background. (a)** The variance of SUVr with slow computed tomography (CT) scanning (SCS) was significantly smaller than that of SUVr with helical CT scanning (HCS) for a 37-mm sphere with 10- and 20-mm amplitudes (*F*-test: *p* < 0.05). A significant difference was observed when comparing the mean SUVr of 30- and 20-mm motion amplitudes with stationary conditions (Dunnett’s test: *p* < 0.05). **(b)** The variance of SUVr with SCS was significantly smaller than that of SUVr with HCS for a 28-mm sphere with 30-mm amplitude (*F*-test: *p* < 0.05). Significant differences were observed when comparing the mean SUVr of 30- and 20-mm motion amplitudes with stationary conditions (Dunnett’s test: *p* < 0.05).

The SUVr measured in the phantom with an air background and spheres sized 37 and 28 mm are presented as box plots in Figure 2a and b, respectively. The variance of SUVr using the SCS CT technique was significantly smaller than that of SUVr obtained using the HCS approach. Figure 3a and b show box plots of the SUVr of spheres of 37 and 28-mm diameters, respectively, measured in a phantom with water background. The variance in SUVr calculated using the SCS CT technique was significantly smaller than that of SUVr obtained by HCS. Considering SUVr calculated in phantoms with air and water backgrounds together, the SCS CT technique was found to be a more suitable approach for AC, compared with HCS. Consequently, we only analysed SUVr calculated using the SCS CT technique in subsequent experiments.

The effect of motion amplitude on SUVr was then investigated. There was a significant difference between SUVr values obtained with different amplitudes. Conversely, the 10-mm motion amplitude did not affect the SUVr. With motion amplitudes of 20 mm and above, the decrease in SUVr was found to be relative to the amplitude of phantom motion. The largest observed SUVr difference, compared with the SUVr obtained under stationary conditions, was 30.9%, calculated for the 28-mm sphere in the phantom with water background at 30-mm motion amplitude (Figure 3b).

### TH_max_

**Figure 4:**
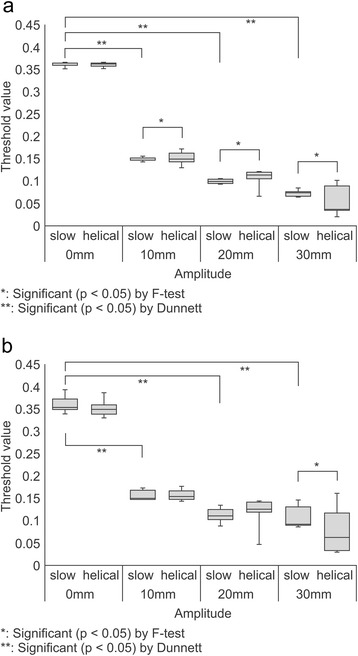
**Effect of motion amplitude on threshold values based on maximum standard uptake values (TH**_**max**_ **), evaluated with air as the phantom background. (a)** The variance of TH_max_ with slow computed tomography (CT) scanning (SCS) was significantly smaller than that of TH_max_ with helical CT scanning (HCS) for a 37-mm sphere with 10-, 20- and 30-mm amplitudes (*F*-test: *p* < 0.05). A significant difference was observed comparing the mean TH_max_ of 30-, 20-, and 10-mm motion amplitudes with stationary conditions (Dunnett’s test: *p* < 0.05). The average TH_max_ value for non-motion was 0.362. **(b)** The variance of TH_max_ with SCS was significantly smaller than that of TH_max_ with HCS for a 28-mm sphere with 30-mm amplitude (*F*-test: *p* < 0.05). A significant difference was observed when comparing the mean TH_max_ of 30-, 20-, and 10-mm motion amplitudes with stationary conditions (Dunnett’s test: *p* < 0.05). The average TH_max_ value for non-motion was 0.352.

**Figure 5:**
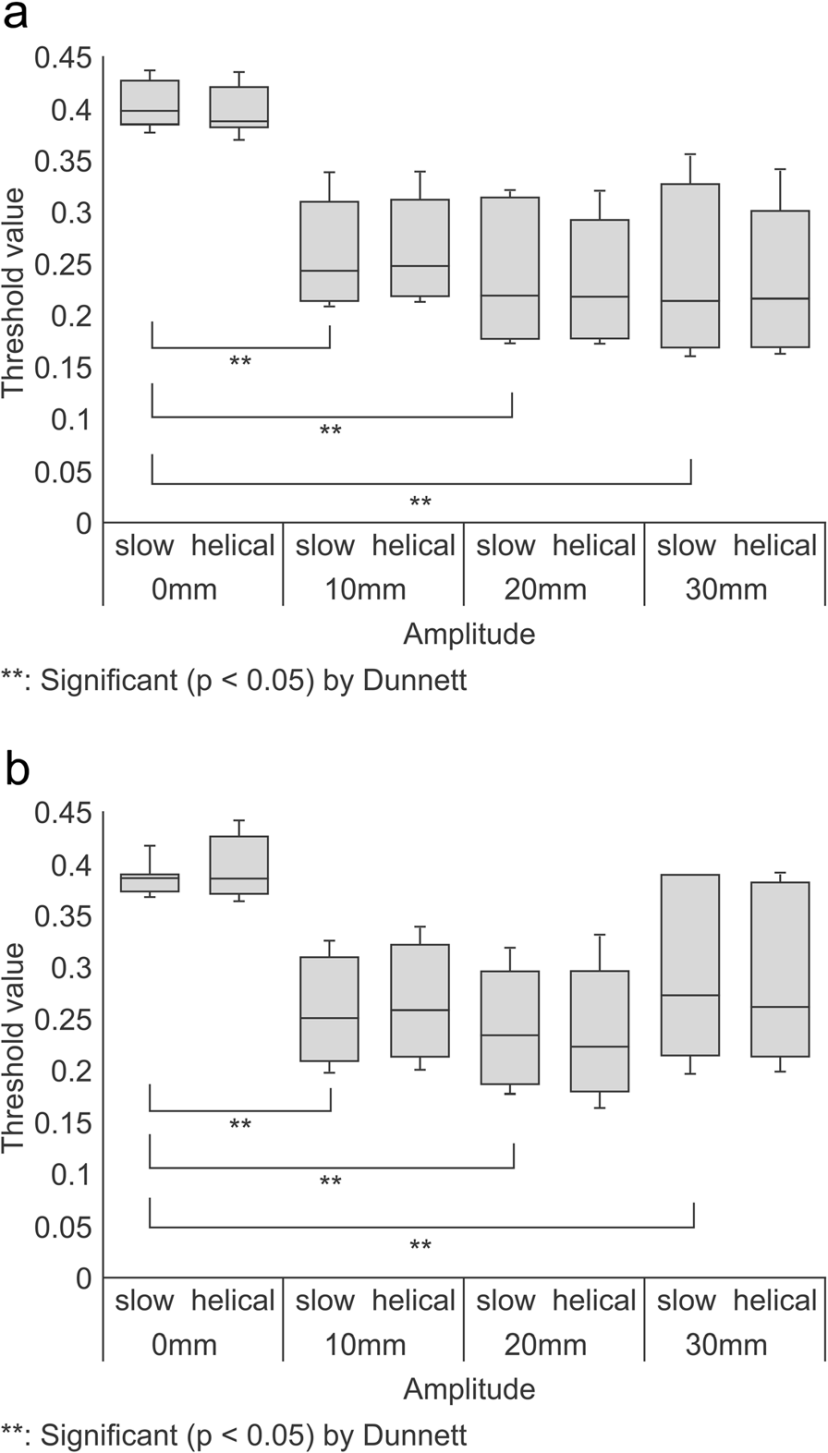
**Effect of motion amplitude on threshold values based on maximum standard uptake values (TH**_**max**_ **), evaluated with water as the phantom background. (a)** For a 37-mm sphere, the variance of TH_max_ did not differ significantly between slow computed tomography (CT) scanning (SCS) and helical CT scanning (HCS). A significant difference was observed between the mean TH_max_ of 30-, 20-, and 10-mm motion amplitudes compared with stationary conditions (Dunnett’s test: *p* < 0.05). The average TH_max_ values for non-motion and motion were 0.404 and 0.244, respectively. **(b)** For a 28-mm sphere, the variance of TH_max_ did not differ significantly between SCS and HCS. A significant difference was observed between the mean TH_max_ of 30-, 20-, and 10-mm, motion amplitudes compared with stationary conditions (Dunnett’s test: *p* < 0.05). The average TH_max_ values for non-motion and motion were 0.387 and 0.263, respectively.

TH_max_ values obtained in the phantom with an air background using 37- and 28-mm spheres are presented as box plots in Figure 4a and b, respectively. The variance of TH_max_ values obtained using the SCS technique was significantly smaller than that obtained using the HCS approach. Figure 5a and b present box plots of the TH_max_ calculated for 37- and 28-mm spheres, respectively, using measurements obtained in a phantom with a water background. For the 37- and 28-mm spheres, the variance of TH_max_ did not differ significantly between SCS and HCS. When we consider the results of TH_max_ for the phantoms with a water or air background together, the use of the SCS method for AC of PET data resulted in less variance, compared with the HCS approach. Therefore, in subsequent experiments we only analysed the TH_max_ values derived from PET data with AC utilizing the SCS method.

**Figure 6:**
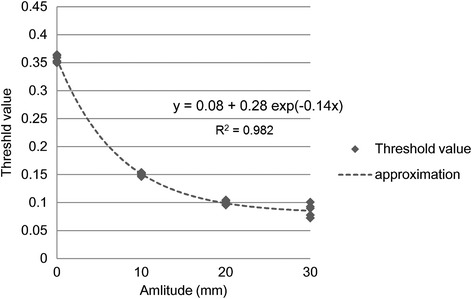
**Threshold values (TH**_**max**_ **) obtained at a range of motion amplitudes measured in a phantom with an air background.** Regardless of concentration or size, TH_max_ was dependent only on amplitude. Because the data fit well (R^2^ = 0.982) with an exponential function, it may be possible to use the function to approximate TH_max_ from motion amplitude.

The effect of motion amplitude on TH_max_ was then investigated. We observed a significant difference between TH_max_ values obtained at different amplitudes. For spheres of 37- and 28-mm diameters in the phantom with an air background, significant differences were compared with the stationary conditions (Figure 4a and b). The average TH_max_ values for 37-mm and 28-mm spheres were 0.362 and 0.352 in non-motion, respectively. For 37- and 28-mm spheres with the water background phantom, significant differences were individually compared with the stationary conditions (Figure 5a and b). The average TH_max_ value for 37-mm and 28-mm spheres were 0.404 and 0.387 in non-motion, respectively. Moreover, the average TH_max_ value for 37-mm and 28-mm spheres were 0.244 and 0.263 in motion, respectively. When the phantom background was filled with air, regardless of the sphere concentration or size, TH_max_ was dependent only on the motion amplitude. Therefore, it may be possible to approximate TH_max_ using the following formula (Figure 6):

4 $$ \mathrm{y}=0.08+0.28\  \exp \left(-0.14\mathrm{x}\right), $$

where y is the approximate value of TH_max_, and x is the motion amplitude.

The largest TH_max_ deviation from the stationary conditions was 82.0%, calculated for the 37-mm diameter sphere with an air background phantom and 30-mm motion amplitude (Figure 4a). When the phantom background was water, no relation between the decrease in TH_max_ and amplitude was observed. At amplitudes greater than 10 mm, TH_max_ was reduced in comparison to the stationary conditions (Figure 5a and b).

## Discussion

In this study, we aimed to extract ITV-b from PET images using calculations from phantom studies. The results of the current study have demonstrated that tumour size, motion amplitude, tumour site (lung or head-and-neck, abdomen), and AC can have a significant impact on PET/CT imaging outcomes, including SUV_max_ and TH_max_. These four factors are individually discussed below.

In considering tumour size, we found no influence of PVE in the experiments with 28- and 37-mm spheres. In spheres with a diameter of 22 mm or less, reduced SUV estimates can result from PVE. Because the underestimation of SUV is exacerbated by respiratory movement, one should be careful when evaluating targets smaller than 28 mm by estimating the SUV. In cases with volumes larger than spheres of 37-mm diameter, the potential influence of PVE remains to be determined.

When evaluating the effect of target motion amplitude, we observed no SUVr underestimation for 10-mm respiratory motion. In the case of amplitudes of 20 mm or more, SUVr decreased in an amplitude-dependent manner. Moreover, differences in background affected calculated volumes. When the background was air, a significant amplitude-dependent difference in TH_max_ was observed. When the background was water, there was no significant effect of amplitude on TH_max_. With amplitudes greater than 10 mm, the TH_max_ was reduced compared with the stationary condition. TH_max_ was found to be more strongly affected by motion than SUV_max_. Because SUV_max_ is the value of a certain point in an ideal phantom volume, the impact of movement on it is less severe than the impact that movement has on TH_max_.

When considering the differences between simulated tumour sites (lung or head-and-neck, abdomen), head-and-neck and abdominal lesions were less susceptible to error caused by AC because the attenuation factors were uniform throughout water. In contrast, lung lesions may be expected to be more susceptible to errors arising from AC differences because of large density differences between sphere phantoms and air. Using air as the phantom background, regardless of RC or size, TH_max_ was dependent only on amplitude. Therefore, in this context, it was possible to approximate TH_max_ using the equation derived from the cubic equation fit to the data (Eq. 4). Using water as the background for the phantom, TH_max_ was dependent on RC, size, and amplitude. When the average threshold of width in the value is selected, the error margin is included in the extraction capacity. However, we believe that this will become the standard for determining ITV-b.

When we consider differences between ACs, the variance of SUVr and TH_max_ with SCS was not significantly different than that of SUVr and TH_max_ with HCS with water as the phantom background. However, when the phantom background was air, the variance of SUVr and TH_max_ with SCS was significantly smaller than that of SUVr and TH_max_ with HCS. In evaluating the response to therapy, or for planning radiotherapy treatment using PET images, SUV and extracted volumes must not change between scans. Our data suggest that the SCS method provides superior AC in comparison to HCS.

Past phantom experiments indicate variation in the value of TH_max_, although they have been reported 40-50% in the non-motion and 25-35% in the moving phantoms [6-9]. TH_max_ for tumours in different areas has also been reported. They are 15-50% for lung tumours [22,23], 40-50% for head-and-neck tumours [24], and 40% pelvic tumours [25]. In summary, when ITV-b is extracted, the approximate expression based on motion amplitude can be used in the lung field. The approximate expression can be used by understanding the amount of the breath movement by the fluoroscope image. Because TH_max_ has a width with the same amplitude in the head-and-neck and the abdomen, it is difficult to compute a concrete threshold value. However, we believe that extracting BTV using the mean value of TH_max_ may aid in treatment planning. As for the value of TH_max_, 0.40 is the standard value in head-and-neck and lower abdominal areas without respiratory movement. Moreover, 0.25 is the standard value in upper abdominal areas with respiratory movement. However, these threshold values resemble those reported previously and become the first choice, it will be necessary to check the extracted volume.

## Conclusions

In this study, we demonstrate concrete conditions to set ITV-b. There was no PVE with tumour sizes ≥ 28 mm, and the variation between SUV_max_ and TH_max_ may be reduced by using SCS for AC. There was no decrease in respiratory movement from approximately 10 mm for SUV_max_. As for the value of TH_max_, 0.40 became the standard value in areas without respiratory movement in the head-and-neck and the lower abdominal area, and 0.25 became the standard value in upper abdominal areas with respiratory movement. The approximate expression based on the motion amplitude may be adjusted in the lung area.

## Abbreviations

PET: Positron emission tomography
CT: Computed tomography
GTV: Gross tumour volume
PVE: Partial volume effect
SUV: Standardized uptake value
BTV: Biological target volume
ITV: Internal target volume
AC: Attenuation correction
RC: Radioisotope concentration
Rco: Recovery coefficient
SUV_max_: Maximum standardized uptake value
SUVr: Ratios of SUV_max_
TH_max_: Threshold values based on SUV_max_
NEMA: National Electrical Manufacturers Association
IEC: International Electrotechnical Commission
^18^F-FDG: 2-[fluorine-18] fluoro-2-deoxy-D-glucose
SCS: Slow computed tomography scanning
HCS: Helical computed tomography scanning
ANOVA: Analysis of variance
